# Molecular characterization of Ribosomal DNA (ITS2) of hard ticks in Iran: understanding the conspecificity of *Dermacentor marginatus* and *D. niveus*

**DOI:** 10.1186/s13104-020-05326-5

**Published:** 2020-10-09

**Authors:** Parisa Soltan-Alinejad, Zahra Ramezani, Hamideh Edalat, Zakkyeh Telmadarraiy, Farrokh Dabiri, Hassan Vatandoost, Mohammad Ali Oshaghi, Mehdi Mohebali, Seyyed Javad Seyyed-Zadeh, Zabihollah Zarei, Haleh Hanifian, Faham Faghihi, Mandan Abolhasani, Mulood Mohammadi Bavani, Jalil Musavi, Olle Terenius, Ali Reza Chavshin

**Affiliations:** 1 Social Determinants of Health, Research Center, Urmia University of Medical Sciences, Urmia, Iran; 2 Department of Medical Entomology and Vector Control, School of Public Health, Urmia University of Medical Sciences, Urmia, Iran; 3 Department of Medical Entomology and Vector Control, School of Public Health, Tehran University of Medical Sciences, Tehran, Iran; 4 Department of Infectious Diseases, School of Medicine, Urmia University of Medical Sciences, Urmia, Iran; 5 Department of Ecology, Swedish University of Agricultural Sciences (SLU), Uppsala, Sweden

**Keywords:** Ixodidae, Molecular systematics, *Dermacentor*, ITS2, COI

## Abstract

**Objectives:**

Hard ticks (Acari: Ixodidae) are ectoparasites of medical and veterinary importance. They are obligate blood-feeding vectors with the ability to transmit a wide variety of pathogens. Standard morphological keys are normally used for the identification of tick species. However, considering the importance of accurate species identification and the determination of bio-ecological characteristics of species, relying on morphological keys alone can be questionable. In this study, two DNA fragments (ITS2 and COI) were selected for phylogenetic evaluation of Iranian hard tick species belonging to the genera *Dermacentor*, *Hyalomma*, and *Rhipicephalus.*

**Results:**

1229 specimens of *Dermacentor marginatus*,* D. niveus*,* Hyalomma anatolicum*, *Rhipicephalus bursa*, and *R. sanguineus* *s.l* constituting 11 populations were collected from three different climatic and zoogeographical zones in Iran. Morphological studies revealed notable differences in important morphological characteristics between different populations of *D. marginatus*.

The results of ITS2 sequence analysis provided additional evidence which supports the conspecificity of *D. niveus* and *D. marginatus.* Contrary to this finding, the sequence analysis of COI and phylogeny favored the separation of the two species. Given the greater importance of COI in identifying and discriminating species, a possibility heterospecificity between the two species should be considered.

## Introduction

Hard ticks are obligate blood-feeding ectoparasites of medical and veterinary importance. They transmit a wide range of bacterial and viral diseases such as ehrlichiosis, Lyme disease, Crimean Congo hemorrhagic Fever (CCHF), tularemia and anaplasmosis [1]. Considering the importance of species identification and the determination of bio-ecological characteristics of species, there is a need for thorough investigations on the taxonomic and phylogenetic relationships among hard-tick taxa. Morphological methods have been widely used for these purposes, but in some species relying solely on morphological methods may be questionable [2]. Molecular methods, which focus on DNA sequence differences, seems to provide a better tool for the assessment of variation within and between species [3, 4]. There is a rapid evolution of the ITS regions in the tick genome as compared to coding regions. For this reason, ITS regions have been exploited for the discrimination between closely related taxa [5]. Many molecular studies have used this region for the identification of different tick taxa [6–10]. Also, there is a high degree of hyper-variability in the second internal transcribed spacer (ITS 2) which can discriminate different populations of the same species [11, 12].

The status of the species *D. niveus* remains debatable among researchers [13]. *D. marginatus* complex comprises of *D. marginatus*, *D. niveus*, *D. nuttalli*, *D. silvarum*, and *D. ushakovae*. Previous studies have described the discrimination between these species as difficult. Some researchers argue that *D. niveus* is a synonym of *D. daghestanicus*, whereas some others believe in the heterospecificity of *D. niveus*,* with D. daghestanicus* as its junior synonym [14]. Some authors also consider both *D. daghestanicus* and *D. niveus* as valid species [15], but this opinion has not been generally accepted [2]. Moreover, molecular studies of the ITS2 fragment have suggested a conspecificity of *D. marginatus* and *D. niveus *was [16], but other authors still consider these two species as valid, separate species (e.g. [2]). Thus, the validation of the suggestion of conspecificity of these species awaits results from further studies.

The molecular characterization of Ixodidae ticks is of great public health importance, and the application of the ITS2 fragment for tick phylogeny remains a valuable tool for species discrimination. In the present study, 10 populations of four species belonging to three genera of Iranian hard ticks were characterized using the ITS2 fragment. Also, the possibility of the conspecificity of *D. niveus* and *D. marginatus* was examined in the present study using proper phylogenetic analysis of both ITS2 and COI fragments.

## Main text

### Materials and methods

#### Study area

Tick samples were collected from 5 provinces across Iran: Ardebil, Kerman, Tehran, Isfahan, and the Kurdistan Province (Additional file 1: Figure S1).

#### Sample collection and identification

Animal dwellings were visited and Ixodidae ticks were collected seasonally from the abdomen, neck, throat and the legs of hosts (sheep, cows, goats and dogs) using forceps so as not to harm the ticks. The tick specimens were placed inside a collection tube [17]. Information such as collection site, date, humidity and temperature of collection sites, and livestock owner’ name were recorded for each specimen. Samples were identified to species level using standard morphological keys [18, 19]. For the morphological differentiation of *D. marginatus* and *D. niveus*, the keys of [13, 20] were used.

#### Genomic DNA extraction and PCR reactions

Samples were frozen in liquid nitrogen and homogenized. Genomic DNA was extracted from tick samples individually using AccuPrep^®^ Genomic DNA Extraction Kit (Bioneer, South Korea), according to the manufacturer manual. The extracted DNA from each species was subjected to PCR reactions using super PCR mastermix^®^ (Yekta Tajhiz Azma, Iran) and the primers: DITS2-F, 5′-GTGCGTCCGTCGACTCGTTTTGA-3′ and DITS2-R, 5′-ACGGCGGACTACGACGGAATGC-3′ [12], in order to amplify the ITS2 region. The amplification condition for the ITS2 region was as follows: initial denaturation at 95 °C for 5 min followed by 30 cycles of [95 °C for 30 s, 49.5 °C for 30 s, and 72 °C for 30 s] and a final extension at 72 °C for 5 min [11].

Also the COI fragment was amplified using the universal primers below: Forward: 5′-GGAGGATTTGGAAATTGATTAGTTCC-3′ and Reverse: 5′-CCCGGTAAAATTAAAATATAAACTTC-3′ [21]. The PCR conditions for COI amplification were set as follows: initial denaturation at 94 °C for 5 min; followed by 30 cycles of [94 °C for 30 s, 48 °C for 30 s, 72 °C for 30 s] and a final extension at 72 °C for 7 min.

All the amplicons were sequenced (Bioneer Co., South Korea) and the results were analyzed using BLAST search (https://www.ncbi.nlm.nih.gov).

#### Phylogenetic analysis

The phylogeny of the ticks in the present study was deduced by using the Maximum Likelihood method based on the Tamura-Nei model [22]. Initial tree(s) for the heuristic search were obtained automatically by applying Neighbor-Join and BioNJ algorithms to a matrix of pairwise distances estimated using the Maximum Composite Likelihood (MCL) approach, and then selecting the topology with superior log likelihood value. A discrete Gamma distribution was used to model evolutionary rate differences among sites [5 categories (+G, parameter = 2.0513 for the three genera of Ixodidae ticks and +G, parameter = 0.0500 for the *Dermacentor* comparative analysis)]. The MEGA6 software was used for the molecular evolutionary analyses [23]. A bootstrap test including 1000 replicates was performed for each tree and the values (expressed as percentages of 1000 replications) are shown at branch points.

### Results

**Fig. 1:**
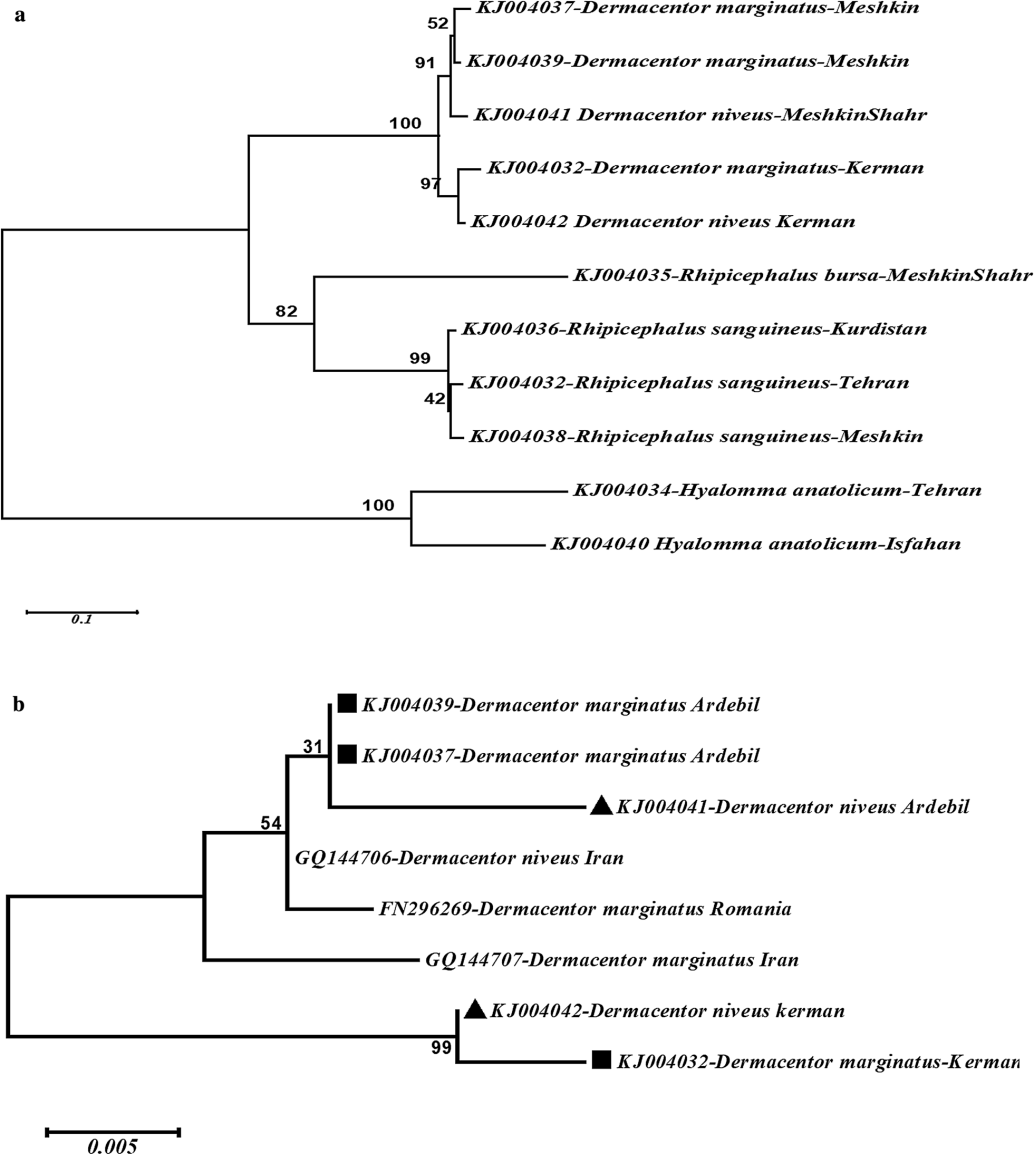
**a** Phylogenetic relationships based on DNA sequences of 638 bp of ITS2 rDNA region for 11 populations of 5 species belonging to 3 genera of hard ticks. For the species names and abbreviations: D: *Dermacentor*, R: *Rhipicephalus*, H: *Hyalomma*, and Mesh (specimens from Ardebil Province, Meshkin-Shahr, The: Tehran Province, Ker: Kerman Province, Kurd: Kurdistan Province and Isf: Isfahan Province). **b** Phylogenetic relationship between the different populations of *D. marginatus* and *D. niveus*. The acquired sequences of these species have been indicated with black square for *D. marginatus* and up-pointing triangle for *D. niveus*

In this study, 1229 specimens of *Dermacentor marginatus*,* D. niveus*,* Hyalomma anatolicum*,* Rhipicephalus bursa* and *R. sanguineus* *s.l* constituting 11 populations were collected from three different climatic and zoogeographical zones in Iran, and the specimens were investigated using molecular tools (Table1). Phylogenetic analysis of the amplified fragments showed that ITS2 sequences can be used to differentiate the three genera investigated in the present study (Fig. 1).

**Table 1:** The species, collection sites, climatic zone and the accession numbers of their ITS2 fragment

Species	Location	Climatic zone	Accession no	Collected samples	
				n	%
*Dermacentor marginatus*	Kerman	Tropical/Desert	KJ004032	980	79.7
	Meshkin-Shahr	Mediterranean/mountainous	KJ004037		
	Meshkin-Shahr	Mediterranean/mountainous	KJ004039		
*Dermacentor niveus*	Meshkin-Shahr	Mediterranean/mountainous	KJ004041	11	0.9
	Kerman	Tropical/desert	KJ004042		
*Rhipicephalus sanguineus s.l*	Tehran	Subtropical/plain	KJ004033	18	1.5
	Kurdistan	Mediterranean/mountainous	KJ004036		
	Meshkin-Shahr	Mediterranean/mountainous	KJ004038		
*Rhipicephalus bursa*	Meshkin-Shahr	Mediterranean/mountainous	KJ004035	195	15.9
*Hyalomma anatolicum*	Tehran	Subtropical/plain	KJ004034	25	2.0
	Isfahan	Subtropical/plain	KJ004040		
Total				1229	100

Similarly, the current protocol using ITS2 could clearly differentiate the two populations of *H. anatolicum*. Also, the current protocol was able to discriminate the members of *Dermacentor* from the rest of the genera, but it could not clearly discriminate between the species of this genus (*D. marginatus* and *D. niveus*) (Fig. 1a).

**Fig. 2:**
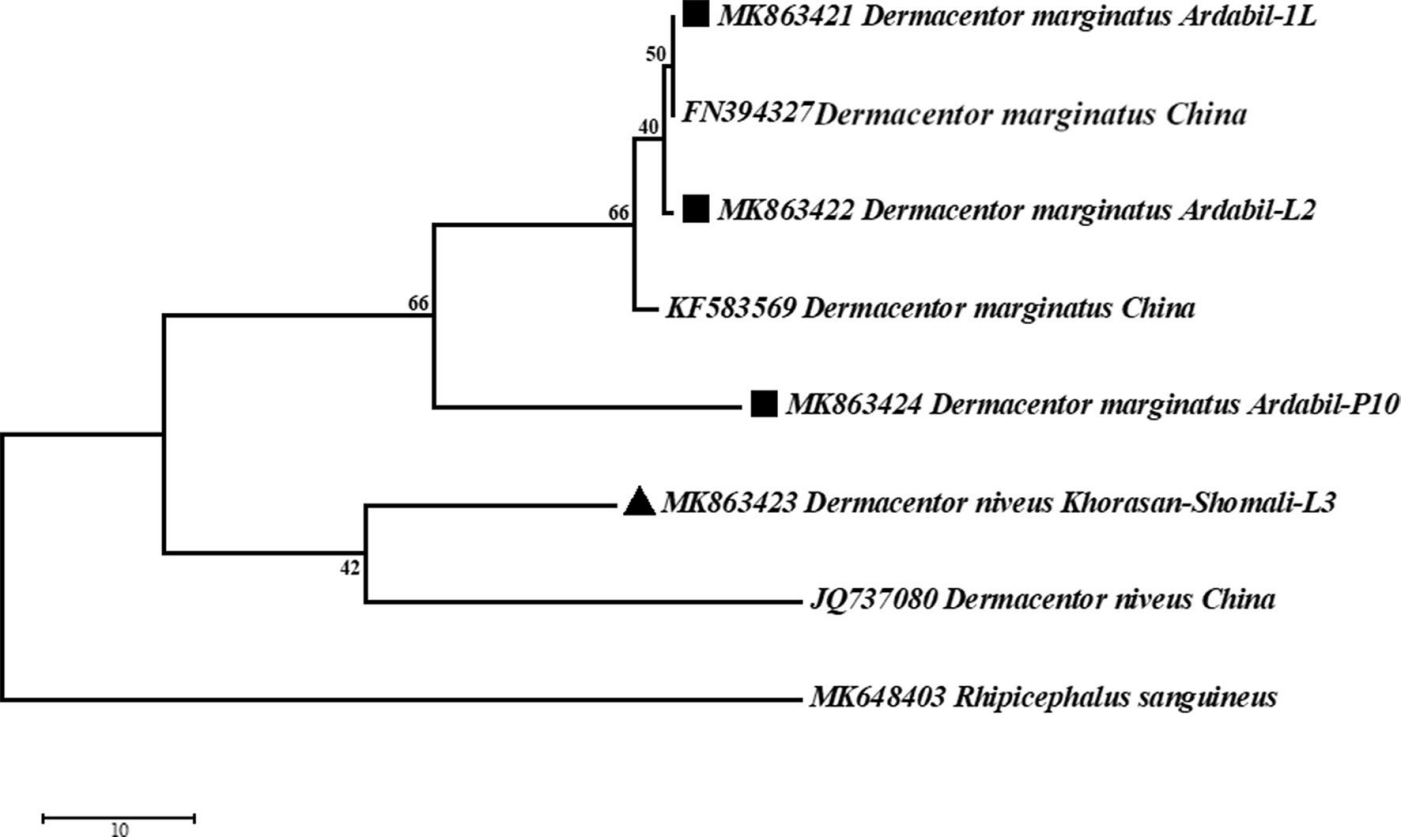
Phylogenetic relationships for DNA sequences of 643 bp of cytochrome Oxidase I (COI) region among different populations of *D. marginatus* and *D. niveus*. (black square indicates the sequences of *D. marginatus* acquired from present study, KF583569 [26] and FN394327 [27] from China, also up-pointing triangle indicates the sequences of *D. niveus*, acquired in current study and JQ737080 from China)

For further analysis of the phylogenetic relationship between the different populations of *D. marginatus* and *D. niveus*, the sequences of these species obtained in our study and from other related studies were analyzed (Fig. 1b). The phylogenetic analysis of the different populations of both *D. marginatus* and *D. niveus* revealed clearly that the ITS2 sequences clustered according to location rather than to species such that *D. marginatus* and *D. niveus* collected from Kerman formed their own clade outside the populations of *D. marginatus* and *D. niveus* from Ardebil. Phylogenetic analysis of COI fragment between the sequences obtained from the present study and the sequences obtained from Gene bank for the two species (*D. marginatus* and *D. niveus*) showed that these species are in two distinct clades. Also, results of the analysis of the COI fragments suggest that these species can be considered as two distinct species (Fig. 2).

### Discussion

This study is one of the few studies that have focused on the molecular characterization of Ixodidae ticks in Iran. Previously, the ITS2 fragments of different populations of the two species of hard ticks (*R. sanguineus s.l* and *D. niveus*) have been characterized in Iran [11, 16].

Based on the results obtained in our study, which confirm that of earlier studies, it can be safely said that ITS2 is a suitable molecular marker at the genus level for distinguishing the examined genera (*Dermacentor*,* Hyalomma* and *Rhipicephalus*). In addition to the identification at the genus level, the results of this study also indicate that this fragment can be successfully used to discriminate between different species. These results also reinforce the suggestion that ITS2 could be used as a standard DNA barcode in the case of a malfunctioned cytochrome oxidase I (COI) fragment.

The ITS2 marker has been shown to have acceptable results in identifying and examining mosquitoes of different populations within a species, and also, it has shown acceptable ability in the identification of ticks at the species level.

In the recently published lists of ticks, the taxonomic position of some species and the validity of their names have generated controversies among experts [2, 24]. The species *D. marginatus* and *D. niveus* were previously distinguished solely based on morphological evaluations, but the limitation of the morphological characters is a major concern [16]. Also, comparison of morphological characteristics and examination of the syntype series of *D. niveus*, revealed that *D. niveus* is conspecific with *D. marginatus* [13]. Similar to this finding, the results obtained in the present study indicate a very high similarity (> 98%) between *D. marginatus* and *D. niveus*, based on the analysis of sequence of different populations of both species obtained in our study and from previous studies in Iran [16] and elsewhere [25] (Fig. 2).

On the other hand, analysis of the COI-barcode, which is regarded as a reliable marker for species identification, identified the two species as heterospecific, valid species. This adds to the complexity of identifying the actual status of the taxonomy of these two species.

The results of the analysis of COI fragment in the present study support a recently published data which suggested that *D. marginatus* and *D. niveus* are different species [2].

## Limitations

In the present study, specimens were collected from limited areas. Doing similar studies over a wide geographical area can produce more reliable results. Accordingly, few specimens of *D. niveus* were collected and studied in the present study. Collection of more specimens of this species from more diverse geographical locations can provide a better picture of the genetic diversity of this species.

## Supplementary information

**Additional file 1: Figure S1.** The map of Iran indicating the collection sites of specimens, 1: Ardebil Province (Meshkin-Shahr), 2: Kurdistan Province, 3: Tehran Province, 4: Isfahan Province and 5: Kerman Province. (Base map has been provided by d-maps.com which is freely available).

## Data Availability

The datasets used and/or analyzed during the current study are available from the corresponding author on reasonable request.

## Abbreviations

ITS2: Internal transcribed spacer 2
COI: Cytochrome oxidase 1
