# Supplementary figures and images for: Molecular characterization of Ribosomal DNA (ITS2) of hard ticks in Iran: understanding the conspecificity of Dermacentor marginatus and D. niveus
Source: BMC Res Notes. 2020 Oct 9;13:478. doi: 10.1186/s13104-020-05326-5 (PMC7547439; doi:10.1186/s13104-020-05326-5)

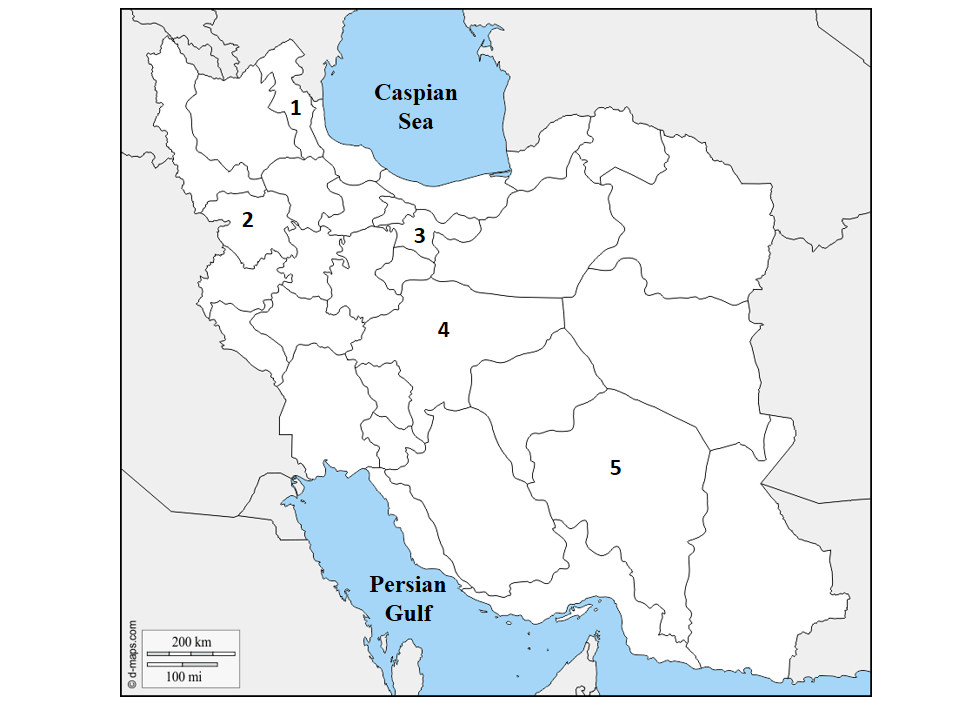

Supplement: Supplementary file 1 — Additional file 1: Figure S1. The map of Iran indicating the collection sites of specimens, 1: Ardebil Province (Meshkin-Shahr), 2: Kurdistan Province, 3: Tehran Province, 4: Isfahan Province and 5: Kerman Province. (Base map has been provided by d-maps.com which is freely available). [file 13104_2020_5326_MOESM1_ESM.tif]
